# The Effect of 5-in-1 Dextrose Neural Prolotherapy for Periscapular Myofascial Pain Syndrome: A Retrospective Study

**DOI:** 10.7759/cureus.98137

**Published:** 2025-11-30

**Authors:** Ashish Yadav, Shalu Arimbooth, Swapnil Sonune, Harshanand Popalwar, Rohit R Gaikar, Sonali Sahani

**Affiliations:** 1 Physical Medicine and Rehabilitation, All India Institute of Medical Sciences, Nagpur, Nagpur, IND

**Keywords:** dextrose injection, dorsal scapular nerve, myofascial pain syndrome, neural prolotherapy, pain management, periscapular pain, spinal accessory nerve, trigger point

## Abstract

Background and objective

Myofascial pain syndrome (MPS) is a chronic musculoskeletal condition characterized by the presence of myofascial trigger points (MTrPs) that cause localized and referred pain, often leading to functional impairments. Despite a multimodal therapeutic approach, some cases prove refractory to conservative management. This study aimed to evaluate the effectiveness of a 5-in-1 dextrose neural prolotherapy injection targeting key muscles and nerves in the periscapular region among patients with chronic MPS.

Methods

A retrospective case series was conducted involving 16 patients with chronic periscapular MPS persisting for at least six months. The 5-in-1 dextrose neural prolotherapy injection involved ultrasound-guided administration of dextrose, lignocaine, and saline to the trapezius, rhomboid minor, levator scapulae, and hydrodissection of the spinal accessory and dorsal scapular nerves (DSN). Pain severity was assessed using the Numerical Rating Scale (NRS) at baseline and three months post-injection.

Results

Sixteen patients (four males, 12 females; mean age: 35.75 ± 12.76 years) were included. The mean NRS pain score decreased significantly from 7.06 ± 0.85 at baseline to 3.25 ± 2.26 at three months post-intervention (mean reduction: 3.81; 95% confidence interval (CI): 2.63-4.99; p<0.001). No statistically significant differences in pain reduction were observed between genders. No major complications were reported.

Conclusions

The 5-in-1 dextrose neural prolotherapy injection resulted in a significant reduction in pain intensity for patients with chronic periscapular MPS refractory to conservative management. These results highlight the need for larger, controlled studies to validate its effectiveness and refine treatment protocols.

## Introduction

Myofascial pain syndrome (MPS) is a chronic condition characterized by recurring pain in the muscles, fascia, or associated soft tissues. It is often accompanied by emotional disturbances or functional impairments [1]. A hallmark of MPS is the presence of myofascial trigger points (MTrPs) - hypersensitive spots within taut bands of muscle that produce localized as well as referred pain. Clinical studies have shown that at least 40.0% of skeletal muscle pain syndrome is mainly caused by the activated trigger points in painful muscles [2]. Although robust population-based data are limited, epidemiological evidence suggests a substantial clinical burden. A pain-centre survey reported that MTrPs were the primary source of pain in approximately 30% of patients presenting to primary care and in up to 85% of those attending tertiary pain clinics [3]. MPS most commonly affects the neck, shoulders, and back. The concept of myofascial pain was first introduced by American physician Dr. Janet Travell in 1952 [4]. Since then, the condition - also referred to as myofascitis, myofascial fibrositis, myositis, fibromyositis, muscle strain, or myofascial syndrome - has gained increasing recognition among clinicians and researchers alike [4].

Myofascial pain may occur after an injury, with chronic strain from repetitive microtrauma, or without any clear precipitating event. Aberrant body mechanics or postural abnormality may trigger the condition or contribute to its persistence [5]. The quality of pain tends to be a deep “aching” of variable intensity, and the pain is generally confined to a specific anatomic region. Characteristic referred pain patterns are associated with particular muscles, although these referral patterns are often unreliable [6]. While the diagnosis of MTrPs remains a topic of debate, Simons et al. have proposed a widely accepted set of clinical criteria [7]. The minimum diagnostic criteria include the presence of a palpable taut band within a skeletal muscle, a hypersensitive spot within that band, and reproduction of the patient’s typical referred pain upon stimulation. Confirmatory findings may include a local twitch response, jump sign, patient recognition of the elicited pain (for active MTrPs), predictable referred pain patterns, muscle tightness or weakness, and pain reproduced with stretching or contraction of the affected muscle [7].

MTrPs may be initiated by an abnormal increase in acetylcholine release at the motor endplate, resulting in sustained muscle contraction. Traumatic or microtraumatic conditions, such as acute or chronic local overload, can further exacerbate this effect [8]. The persistent contraction increases local energy demand while simultaneously reducing blood flow, leading to localized ischemia. These physiological changes may trigger pain or hypersensitivity by promoting the local release of nociceptive substances in the affected area [9].

The effective management of MPS requires identifying and addressing the underlying etiological and perpetuating factors. Without addressing these underlying contributors, therapeutic interventions may yield only temporary relief, and the condition is likely to recur or persist [10]. The treatment involves a multimodal approach, including both conservative and various interventional procedures. Tang et al. have described a 5-in-1 neural prolotherapy injection technique that targets key muscles commonly involved in myofascial pain: the trapezius, rhomboids, levator scapulae, and hydrodissection spinal accessory nerve (SAN) and dorsal scapular nerve (DSN), all through a single percutaneous approach.

Beyond addressing intramuscular trigger points, this technique is also employed with an emphasis on hydrodissection of the SAN and DSN, aiming to relieve pain potentially associated with peripheral nerve entrapment [11]. One proposed mechanism of action suggests that dextrose binds to presynaptic calcium channels and inhibits the release of substance P and CGRP, thereby decreasing neurogenic inflammation [12]. This study aimed to evaluate the efficacy and safety of ultrasound-guided 5-in-1 dextrose neural prolotherapy for the management of chronic periscapular MPS. We describe the clinical outcomes following this multi-target injection technique in 16 patients, focusing on its potential to provide significant pain relief by addressing multiple anatomical pain sources under real-time ultrasound guidance.

## Materials and methods

Patient selection criteria

Patients considered for the 5-in-1 dextrose neural prolotherapy injection technique typically present with chronic periscapular upper back pain lasting six months or more. The pain distribution in periscapular MPS typically involves the upper and middle fibres of the trapezius, rhomboid major and minor, and levator scapulae muscles. It is most often localized along the medial border of the scapula and may radiate toward the ipsilateral posterior shoulder or lower cervical region. Unlike pain arising from cervical radiculopathy or intrinsic shoulder joint disorders, periscapular myofascial pain is primarily muscular in origin. All procedures were performed by a single senior resident physician with a minimum of three years’ experience in musculoskeletal ultrasound-guided pain interventions, and patients consistently demonstrated discrete, palpable trigger points that reproduced their characteristic pain upon compression.

Cervical or shoulder movements may exacerbate discomfort due to muscle tension, but do not produce the neurological deficits or joint-specific mechanical limitations typically associated with spinal or glenohumeral pathology. These patients have generally not responded to a full course of conservative management, including analgesics, muscle relaxants, neuropathic agents (e.g., gabapentin or amitriptyline), and physical therapy targeting stretching and strengthening of the affected muscles, or were unable to engage in rehabilitation programs due to severe pain. Clinical evaluation often reveals palpable myofascial trigger points in the periscapular region, and in some cases, there may be signs suggestive of peripheral nerve entrapment involving the DSN or SAN.

These findings support the use of targeted intervention through the 5-in-1 dextrose neural prolotherapy injection to address both muscular and neural contributors to pain. Patients did not have any active infections at the time of enrollment. Once enrolled, a thorough clinical examination was performed, and baseline pain severity was recorded using the Numerical Rating Scale (NRS). All procedures conducted in this case series were performed following the ethical standards of the institutional and/or national research committees and conformed to the principles of the Declaration of Helsinki (as revised in 2013). Written informed consent was obtained from all patients for inclusion in the case series and for the publication of their clinical data and accompanying images.

Injection technique

**Figure 1:**
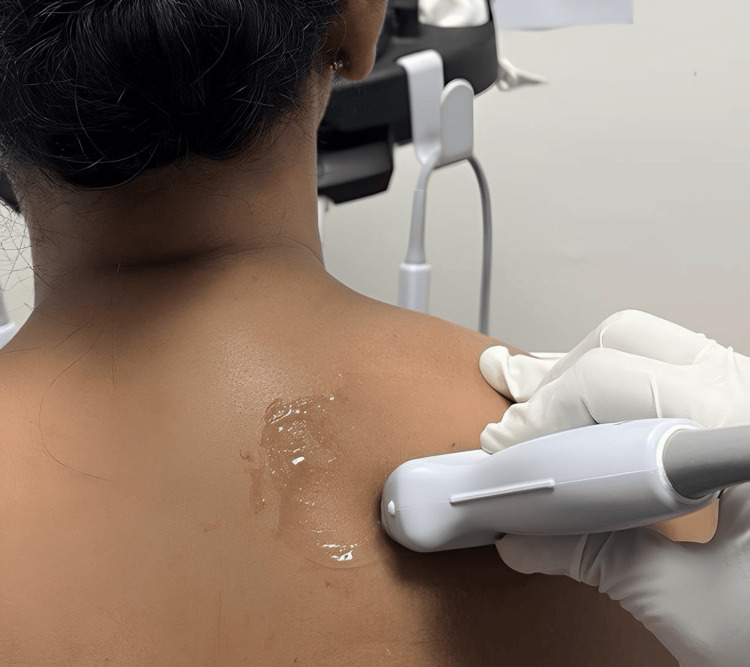
Ultrasound probe at the level of scapular spine in transverse view

With the patient seated upright in a chair or on an examination table, their back was positioned toward the examiner. The medial border of the scapula was palpated, and using our institute’s ultrasound system (Sonosite Edge II, FUJIFILM Sonosite, Bothell, WA) with a high-frequency linear transducer (6-15 MHz), the probe was oriented medially to laterally at the level of the scapular spine (Figure 1).

**Figure 2:**
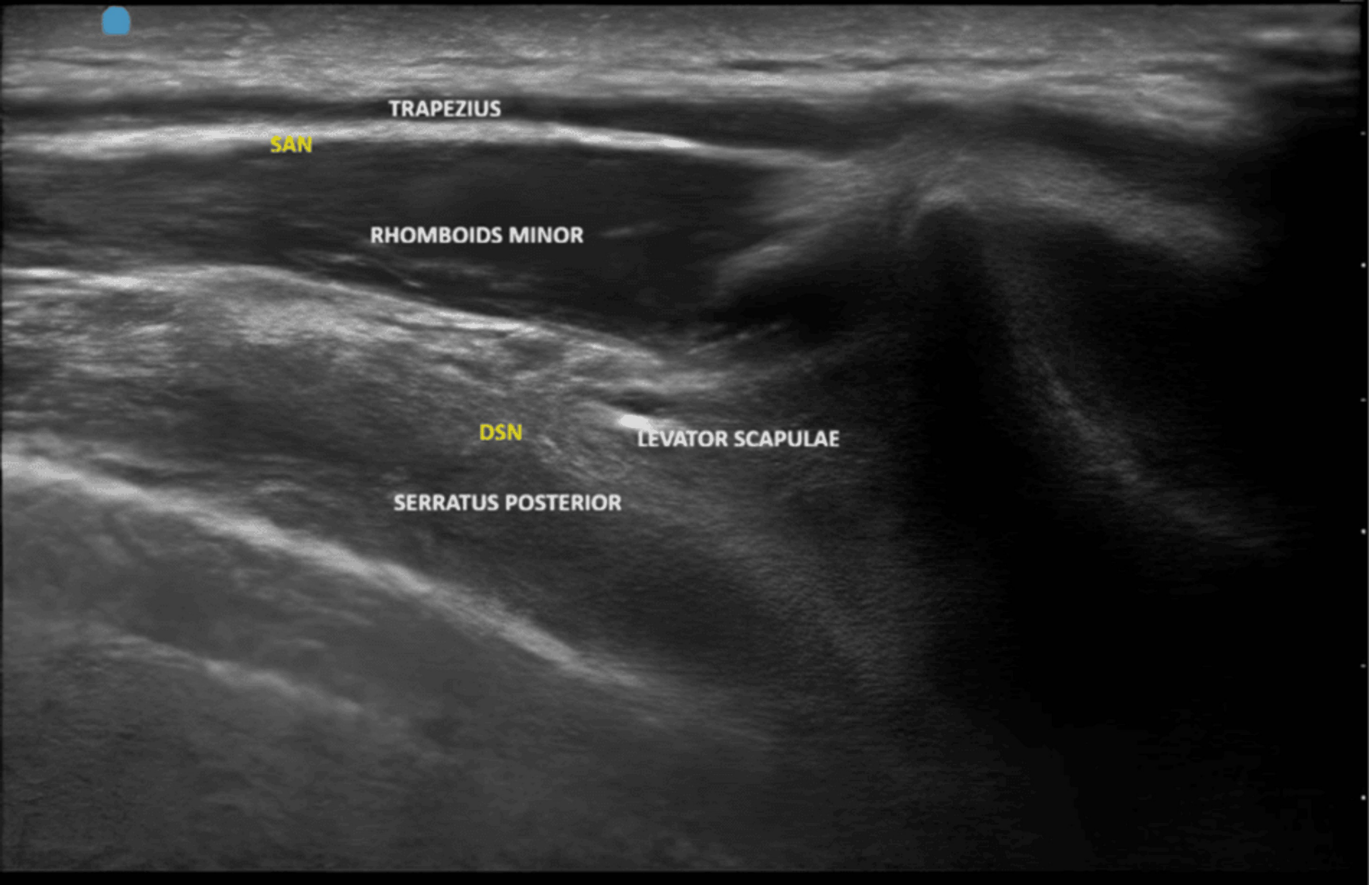
Sonoanatomy of periscapular region SAN: spinal accessory nerve; DSN: dorsal scapular nerve

**Figure 3:**
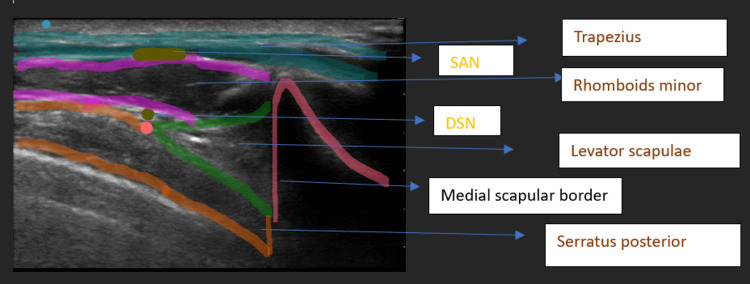
Labelled diagram SAN: spinal accessory nerve; DSN: dorsal scapular nerve

The trapezius muscle was visualized superficially, with the levator scapulae or rhomboid minor seen deeper, depending on the level. The SAN was identified in the fascial plane between the trapezius and rhomboid minor. Deeper structures included the serratus posterior superior, paraspinal muscles, and pleura (Figures 2, 3).

Using Doppler, the dorsal scapular artery (DSA) and accompanying nerve were identified. The transducer was rotated to a cephalad-caudal orientation to visualize the DSA in the long axis. An in-plane injection was then performed under ultrasound guidance.

After local anesthesia of the skin was administered using a 25-gauge, 1.5-inch needle with 2% lignocaine, a 22-gauge, 3.5-inch spinal needle was inserted in a medial-to-lateral direction. A 10 mL solution containing 4 mL of 25% dextrose, 4 mL of 2% lignocaine, and 2 mL of normal saline is used for the injection. The trapezius muscle was infiltrated first, followed by hydrodissection of the spinal accessory nerve. The needle was then advanced to the rhomboid minor muscle, which was subsequently infiltrated. Next, the adjacent DSN was hydrodissected in the fascial plane between the serratus posterior superior, levator scapulae, and rhomboid minor muscles. At the end levator scapulae muscle was infiltrated. Each targeted location was injected with 2 cc of the above solution. Finally, the needle was withdrawn, and the injection site was covered with a bandage. After the procedure, patients were observed for an hour for any immediate complications.

Patients were given standardized post-procedure instructions, which included avoiding the use of nonsteroidal anti-inflammatory drugs (NSAIDs) for at least 48 hours following the injection. For analgesia, local injection site cold compression for 15 minutes - three times/day for two days - and oral acetaminophen was prescribed as needed, with a maximum allowable dose of 3 grams per day. Patients were also advised to begin a structured exercise program focused on gentle stretching and strengthening of the scapular stabilizer muscles, along with cervical and shoulder range-of-motion (ROM) exercises to restore flexibility and prevent recurrence. In addition, ergonomic counseling was provided to promote workplace and postural modifications, with education on how improper ergonomics and repetitive strain may contribute to the perpetuation of periscapular myofascial pain.

Post-procedure follow-up was performed three months after the intervention in the PMR outpatient department. Pain was assessed using the NRS. The collected data were entered and analyzed using IBM SPSS Statistics for Windows, version 29.0 (IBM Corp., Armonk, NY).

## Results

A total of 16 patients clinically diagnosed with MPS were included in this study. The demographic characteristics are summarized in Table 1.

**Table 1:** Demographic details of the participants (N=16) SD: standard deviation

Characteristics		Values
Gender	Male	4
	Female	12
Age, years	Mean (SD)	35.75 (12.76)

Among the participants, four were males, and 12 were females, with a mean age of 35.75 (12.76) years. All patients underwent a 5-in-1 dextrose neural prolotherapy injection targeting relevant peripheral nerves associated with pain generation. Pain outcomes were evaluated using the NRS at baseline (pre-procedure) and three months post-intervention. The mean NRS score at baseline was 7.06 (0.85), which significantly reduced to 3.25 (2.26) at the three-month follow-up (Table 2).

**Table 2:** Comparison of NRS scores across different time points (N=16) ^*^Unpaired t-test, SD: standard deviation; CI: confidence interval

Characteristics	Values
NRS: 0 month, mean (SD)	7.0625 (0.85)
NRS, 3 months, mean (SD)	3.25 (0.56)
Mean difference (95% CI)	3.81 (2.63 - 4.99)
Test statistics	6.8457
P-value*	<0.001

**Figure 4:**
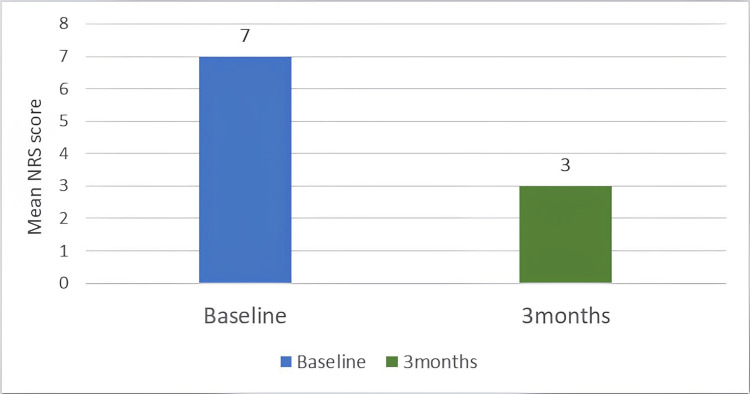
Graphical representation of NRS severity over time Pain severity over time NRS: Numerical Rating Scale

Figure 4 illustrates the NRS severity over time, reflecting a substantial overall reduction in pain intensity.

The mean reduction in NRS was 3.81 (95% CI: 2.63 - 4.99), and the change was statistically significant (t = 6.8457, p<0.001).

**Figure 5:**
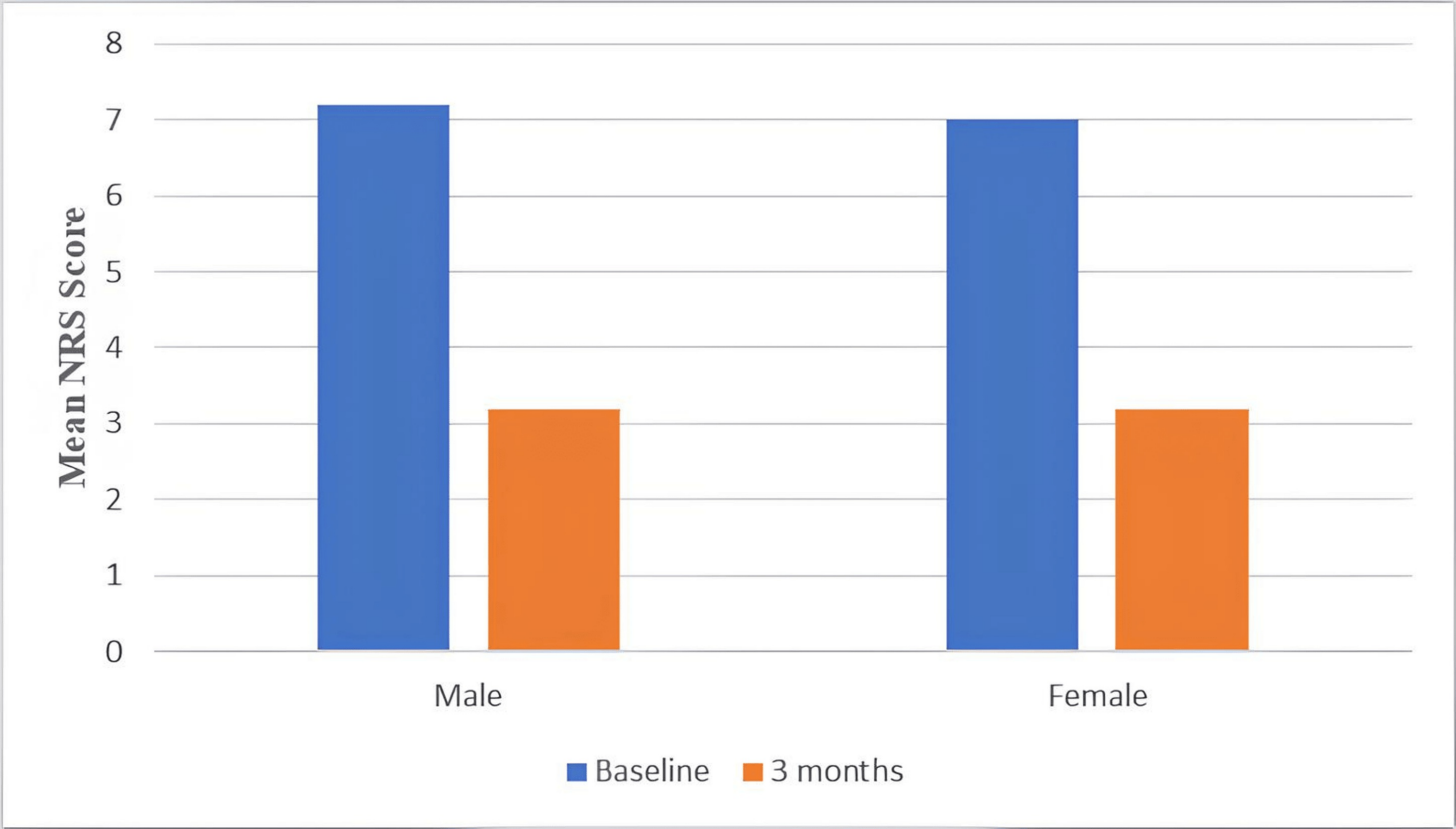
Graphical representation of NRS comparison by gender NRS: Numerical Rating Scale

When analyzed by gender (Table 3, Figure 5), the baseline NRS scores were 7.25 (0.50) for males and 7.00 (0.95) for females.

**Table 3:** Comparison of NRS scores at different time points by gender ^*^Paired t-test NRS: Numerical Rating Scale; SD: standard deviation

Timepoint	Male NRS, mean (SD)	Female NRS, mean (SD)	P-value*
0 month	7.25 (0.5)	7 (0.95)	0.628
3 months	3.25 (2.5)	3.25 (2.3)	1.000

At three months, both genders reported an identical mean NRS score of 3.25, though with varying standard deviations (males: 2.5; females: 2.3). However, no statistically significant differences were observed between genders at either time point (baseline p=0.628; three months p=1.000).

Among the cohort, 10 patients (62.5%) demonstrated a clinically meaningful improvement, defined as greater than 50% reduction in pain scores. Notably, one patient reported complete resolution of pain, with an NRS score of 0, whereas one patient showed no change in pain levels following the procedure. The remaining patients experienced varying degrees of pain relief.

Importantly, no adverse events or post-procedural complications were observed in any of the 16 patients during the follow-up period, highlighting the safety and tolerability of the 5-in-1 dextrose neural prolotherapy injection in this patient population. These findings suggest that this multi-target nerve block approach may be an effective and well-tolerated therapeutic option for individuals suffering from MPS.

## Discussion

Dextrose prolotherapy has been employed in clinical settings for several decades as a regenerative injection therapy aimed at treating various musculoskeletal and myofascial pain syndromes. It involves the injection of a hyperosmolar dextrose solution into affected ligaments, tendons, joint spaces, or myofascial trigger points to stimulate a localized inflammatory response that promotes tissue repair and remodeling. Numerous studies have demonstrated its ability to enhance fibroblast proliferation, collagen synthesis, and overall extracellular matrix production, thereby contributing to the structural integrity and functional recovery of soft tissues [13].

This study investigated the efficacy and safety of ultrasound-guided 5-in-1 dextrose neural prolotherapy in patients with chronic periscapular MPS resistant to conventional treatment. The intervention targeted multiple anatomical structures implicated in pain generation, including the trapezius, rhomboid minor, levator scapulae muscles, and the spinal accessory and dorsal scapular nerves. Baseline pain scores, measured by the NRS, showed a significant and clinically meaningful reduction from 7.06 (0.85) to 3.25 (2.26) at three months post-treatment, with a mean difference of 3.81 points (95% CI: 2.63-4.99; p<0.001). Clinically, more than 60% of patients achieved a ≥50% reduction in pain, with one patient reporting complete pain resolution. There were no adverse events during the follow-up. These results underscore the treatment’s potential effectiveness and safety in a population that had previously failed various conservative therapies.

Several well-controlled studies and reviews have demonstrated that dextrose prolotherapy notably outperforms placebo (e.g., saline injections) in reducing pain and improving function in patients with MPS and other musculoskeletal conditions. A systematic literature review data from 44 case series, two nonrandomized controlled trials (NRCT), and nine randomized controlled trials (RCT) revealed that seven out of nine double-blind, placebo-controlled trials showed statistically significant improvements in pain and/or function with dextrose prolotherapy for conditions including MPS, sacroiliac pain, and tendinopathies (level 1 and 2 evidence) [14]. In a retrospective review by Chou et.al, ultrasound-guided dextrose injection provided significant symptom relief in refractory myofascial pain, with 80% of patients reporting >50% improvement and a 65% mean VAS reduction within one month [15]. The mechanism is thought to extend beyond needle-induced effects, involving modulation of inflammation and tissue repair.

Perineural injection with dextrose has been increasingly utilized to target peripheral nerves implicated as pain generators in various chronic pain syndromes. Unlike traditional prolotherapy, which is proposed to act through stimulation of local tissue repair and proliferation, the mechanism of perineural injection appears to be distinct. Current evidence suggests that its analgesic effect is mediated through modulation of neuropathic pain pathways, including reduction of neurogenic inflammation, stabilization of nerve membranes, and downregulation of abnormal nerve signaling rather than by proliferative effects on connective tissue [16]. This mechanism may explain the growing clinical interest in dextrose as a treatment for neuropathic and myofascial pain conditions, particularly in cases refractory to conventional therapies. Overall, the evidence supports dextrose prolotherapy as a promising, cost-effective, and generally safe alternative to placebo, with efficacy that can rival or even exceed that of other injectables, depending on the context. However, further high-quality randomized trials are needed to clarify comparative long-term outcomes and optimal dosing strategies.

A retrospective review conducted by Modi et al. highlights the potential efficacy and safety of the 5-in-1 nerve block in the management of MPS. Among the 25 patients treated, a significant reduction in pain intensity was observed over three months, with the mean NRS score decreasing from 6.5 at baseline to 4.2 post-procedure. A clinically meaningful improvement, defined as a greater than 40% reduction in pain, was achieved in 40% of the patients after the three-month follow-up [14]. The favorable response in the majority of patients may be attributed to the comprehensive nature of the 5-in-1 block, which simultaneously targets multiple peripheral nerves commonly involved in myofascial pain transmission. Tang et al. have described ultrasound-guided 5-in-1 technique injection for trigger point injections in trapezius, levator scapulae, rhomboid muscle, and nerve hydrodissection of SAN and DSN in single injections for nonspecific upper back pain [11].

In the context of myofascial pain, prolotherapy may provide additional benefit by modulating neurogenic inflammation and improving local circulation, which are often impaired in chronic pain states. The cases presented in this series showed meaningful clinical improvement following dextrose prolotherapy, particularly in patients who had not responded adequately to conventional interventions such as physical therapy, NSAIDs, or trigger point injections. These findings are consistent with emerging evidence supporting the efficacy of prolotherapy as a low-risk, cost-effective treatment option for chronic soft tissue pain.

Further research, including RCTs, is warranted to better elucidate the optimal concentrations, injection techniques, and long-term outcomes associated with dextrose prolotherapy in diverse patient populations. However, our observations suggest that this modality holds promise as an adjunct or alternative in the management of persistent myofascial and musculoskeletal disorders. Importantly, no complications or adverse events were noted among the 16 participants, supporting the safety profile of ultrasound-guided nerve blocks, which allow for precise localization and reduce the risk of iatrogenic injury.

While the results are promising, the small sample size and lack of a control group limit the generalizability of the findings. Nonetheless, this study adds to the growing body of evidence supporting interventional strategies for MPS and provides a basis for future prospective, randomized controlled trials to validate efficacy, determine optimal nerve combinations, and evaluate long-term outcomes.

## Conclusions

The findings from this case series suggest that the ultrasound-guided 5-in-1 dextrose neural prolotherapy is a promising and safe intervention for MPS, with a majority of patients experiencing clinically meaningful pain relief at three months post-procedure. The simultaneous blockade of multiple peripheral nerves may provide broader analgesic coverage and address the complex neuroanatomy underlying MPS, which is often refractory to single-site injections or conventional therapies. Clinically, these results resonate with recent reports and systematic reviews that emphasize the importance of multimodal and region-specific approaches in chronic pain syndromes, especially where trigger point sensitivity and peripheral nerve involvement play a crucial role. The integration of nerve block techniques into interventional pain management aligns with evidence supporting ultrasound guidance for precision, safety, and improved outcomes. However, the current case series is limited by its small sample size, lack of randomization or control group, and short follow-up duration. Furthermore, pain perception and response to interventions are multifactorial, and future research should evaluate psychological, occupational, and functional outcomes in addition to pain scores.

Importantly, no adverse events were noted, echoing published data on the safety profiles of ultrasound-guided and nerve block interventions in similar settings. Nevertheless, high-quality RCTs are required to validate these preliminary observations, establish optimal nerve combinations and injectable concentrations, assess the durability of effect, and explore the long-term impact on quality of life. In summary, this report supports the 5-in-1 dextrose neural prolotherapy as a valuable addition to the interventional armamentarium for refractory MPS and provides a foundation for further research into best practices for nerve block techniques in chronic musculoskeletal pain.
